# Cannabidiol Modulates the Motivational and Anxiety-Like Effects of 3,4-Methylenedioxypyrovalerone (MDPV) in Mice

**DOI:** 10.3390/ijms22158304

**Published:** 2021-08-02

**Authors:** Laia Alegre-Zurano, Raúl López-Arnau, Miguel Á. Luján, Jordi Camarasa, Olga Valverde

**Affiliations:** 1 Neurobiology of Behaviour Research Group (GReNeC-NeuroBio), Department of Experimental and Health Sciences, Universitat Pompeu Fabra, 08003 Barcelona, Spain; laia.alegre@upf.edu (L.A.-Z.); lujanperezma@gmail.com (M.Á.L.); 2 Department of Pharmacology, Toxicology and Therapeutic Chemistry, Faculty of Pharmacy and Food Sciences, Pharmacology Section and Institute of Biomedicine (IBUB), Universitat de Barcelona, 08028 Barcelona, Spain; jcamarasa@ub.edu; 3 Neuroscience Research Programme, IMIM-Hospital del Mar Research Institute, 08003 Barcelona, Spain

**Keywords:** MDPV, cannabidiol, conditioned place preference, self-administration, anxiety, mice

## Abstract

3,4-Methylenedioxypyrovalerone (MDPV) is a new psychoactive substance (NPS) and the most widespread and life-threatening synthetic cathinone of the “bath salts”. Preclinical research has proven the cocaine-like psychostimulant effects of MDPV and its potential for abuse. Cannabidiol (CBD) is a non-psychotropic phytocannabinoid that has emerged as a new potential treatment for drug addiction. Here, we tested the effects of CBD (20 mg/kg) on MDPV (2 mg/kg)-induced conditioned place preference and MDPV (0.05 and 0.075 mg/kg/infusion) self-administration paradigms. In addition, we assessed the effects of the co-administration of CBD and MDPV (3 and 4 mg/kg) on anxiety-like behaviour using the elevated plus maze (EPM). CBD mitigated the MDPV-induced conditioned place preference. On the contrary, CBD administration throughout the MDPV (0.075 mg/kg/infusion) self-administration increased drug-seeking and taking behaviours, but only in the high-responders group of mice. Furthermore, CBD exerted anxiolytic-like effects, exclusively in MDPV-treated mice. Taken together, our results indicate that CBD modulation of MDPV-induced motivational responses in mice varies depending on the requirements of the learning task, resulting in a complex response. Therefore, further research attempting to decipher the behavioural and molecular interactions between CBD and MDPV is needed.

## 1. Introduction

Hundreds of new psychoactive substances (NPS) have been synthesised in the last years [1]. In Europe, synthetic cathinones and cannabinoids account for 77% of all seizures of NPS [2]. The elevated use of synthetic cathinones as “legal highs” has raised concern due to the risk of abuse and dependence. Among them, 3,4-methylenedioxypyrovalerone (MDPV) is one of the most widespread and life-threatening components of the so-called “bath salts” [3,4]. Together with euphoria and enhanced energy, the use of MDPV is related to hyperthermia, dehydration, sweating, loss of appetite, disturbed sleep patterns and consciousness alterations [5]. High doses of MDPV can cause severe symptoms, including hallucinations, psychosis, seizures or even death [6].

MDPV is a potent uptake inhibitor of the dopamine transporter (DAT) and norepinephrine transporter (NET), with weaker activity at the serotonin transporter (SERT) [4,7]. Compared to cocaine, MDPV is 50-fold more potent as a DAT inhibitor [4]. Notably, MDPV administered intravenously (i.v.) increases dopamine in nucleus accumbens and locomotor activity in rats at one-tenth the dose of cocaine [8]. Moreover, MDPV is more potent at increasing locomotor activity and inhibiting DAT than other synthetic cathinones in CD1 mice (Giannotti et al., 2017; Marusich et al., 2014). Consistent with this mechanism of action, some authors have found that MDPV has powerful rewarding effects, measured by ultrasonic vocalisations in rats in the self-administration paradigm [9,10]. In the same line, MDPV induced rewarding effects in the conditioned place preference (CPP) in mice [11] and rats [12,13].

Previous studies reported that MDPV works as a reinforcer during intravenous self-administration in rats [14,15,16,17,18,19,20,21]. However, although Fantegrossi et al. (2013) demonstrated that mice discriminate between MDPV (0.3 mg/kg i.p.) and saline in an operant paradigm [22], MDPV self-administration studies using mice are very scarce. In fact, to date, only one study has reported MDPV (0.3 mg/mL p.o.) self-administration in a two-bottle choice paradigm, showing an escalated oral consumption throughout the day [23]. However, up to now, no study had ever explored MDPV operant self-administration in mice.

Lately, the phytocannabinoid cannabidiol (CBD) has emerged as a potential treatment for neuropsychiatry disorders [24], including anxiety [25], depression [26], and drug abuse [27]. CBD effects are complex due to its wide variety of targets within the central nervous system [28], including its agonism at: 5-hydroxytryptamine 1A, transient potential vanilloid 1, G-protein 55 and peroxisome proliferator-activated gamma receptors; the blockade of adenosine reuptake [28] and the negative allosteric modulation of cannabinoid receptors type 1 and 2 [29,30]. Regarding addiction, the existing literature has pointed out the protective role of CBD for treating psychostimulants abuse [31,32]. Our team reported that CBD (20 mg/kg) decreased cocaine-induced acquisition of CPP in mice [33] and cocaine-maintained self-administration [33,34,35], as well as cocaine-induced drug-seeking behaviour [33]. Similarly, CBD potentiated the extinction of cocaine-induced and amphetamine-induced CPP in rats [36] and methamphetamine-induced drug-seeking behaviour [37].

On this basis, in the present study, we evaluated the effects of CBD on the rewarding and reinforcing effects of MDPV in mice. We first investigated the impact of MDPV on the acquisition of CPP. Then, we examined the reinforcing effects of MDPV at two different doses via the intravenous self-administration and progressive ratio test together with the effects of CBD in such a paradigm. Finally, we assessed the effects of the co-administration of CBD and MDPV on anxiety-like behaviour using the elevated plus maze (EPM) in mice.

## 2. Methods

### 2.1. Animals

Eight-week-old male CD1 mice were purchased (Charles River, Barcelona, Spain) and transported to our animal facility (UBIOMEX, PRBB, Barcelona, Spain). Each mouse only underwent one behavioural test (CPP, *n* = 39; self-administration, *n* = 72; EPM, *n* = 36). Mice that underwent CPP or EPM were maintained in a light-dark cycle, with lights turned off between 19:30 and 07:30. Mice that underwent self-administration were maintained in a reverse light-dark cycle, with lights turned off between 07:30 and 19:30, as this is the circadian phase where they are more active. The UPF/PRBB Animal Ethics Committee (CEEA-PRBB-UPF) approved all animal care and experimental protocols, in accordance with the European Community Council guidelines (2016/63/EU).

### 2.2. Drugs

MDPV hydrochloride was synthesised in our laboratory (IBUB, Universitat de Barcelona), and dissolved in 0.9% NaCl. CBD (20 mg/kgi.p.) was kindly provided by Phytoplant Research S.L., (Córdoba, Spain) and was suspended in a 0.9% NaCl solution containing 2% Tween-80.

### 2.3. Conditioned Place Preference

The test was carried out as previously described [33,38]. The apparatus consisted of two conditioning compartments that differed in visual and tactile cues (30 × 29 × 35 cm) connected by a grey-coloured tunnel (14 × 29 × 35 cm) (Cibertec S.A., Madrid, Spain). Briefly, during the pretest, mice were placed in the central compartment and left free to move along the three compartments for 20 min. During the conditioning phase (4 MDPV pairings, 8 days), mice received an injection of MDPV (2 mg/kg, i.p.) immediately before being placed into one of the two conditioning compartments for 30 min. On alternate days, mice were treated with a saline injection and placed in the other compartment for 30 min. Control animals received saline every day. CBD (20 mg/kg, i.p.) or vehicle was administered during the 8 conditioning days 30 min before the MDPV/saline injection. Twenty-four hours after the conditioning phase, mice were tested in the same conditions as in the pretest. Time spent in the compartment associated with MDPV was measured in the pretest and test as a measure of the degree of conditioning induced by the drug.

### 2.4. Self-Administration

#### 2.4.1. Surgery

The surgical procedure was conducted as previously described [35,39]. Surgical implantation of the catheter into the jugular vein was performed following anaesthetisation with a mixture of ketamine hydrochloride (75 mg·kg^−1^; Imalgène1000, Lyon, France) and medetomidine hydrochloride (1 mg·kg^−1^; Medeson^®^, Barcelona, Spain). Briefly, a 6 cm length of silastic tubing (0.3 mm inner diameter, 0.6 mm outer diameter) (silastic, Dow Corning, Houdeng-Goegnies, Belgium) was fitted to a 22-gauge steel cannula (Semat, Herts, England). The catheter tubing was inserted 1.3 cm into the right jugular vein and anchored with a suture. The remaining tubing ran subcutaneously to the cannula, which exited at the mid-scapular region.

The analgesic meloxicam (0.5 mg/kg s.c.; Metacam^®^, Barcelona, Spain) and the antibiotic enrofloxacin (7.5 mg/kg i.p.; Baytril^®^ 2.5%; Barcelona, Spain) were injected after surgery to counter potential infections. Atipamezole hydrochloride (0.5 mg/kg i.p.; Revertor^®^, Barcelona, Spain) and 1 mL glucose 5% solution were also injected to facilitate recovery from surgery. Home cages were placed on thermal blankets to avoid post-anaesthesia hypothermia. Mice were monitored daily for their weight and treated with meloxicam for 48 h and were allowed to recover for four days before the acquisition phase of the self-administration procedure began.

#### 2.4.2. Acquisition of MDPV Self-Administration

Self-administration experiments were conducted as described previously [40,41]. Mice were trained for 2 h/day to nose poke in order to receive a 0.05 or 0.075 mg/kg/infusion of MDPV on 10 consecutive days, under a fixed ratio 1 (FR1) reinforcement schedule. When mice responded at the active hole, the stimulus light lit up for 4 s and an MPDV infusion was delivered automatically. Each infusion was followed by a 15-s time-out period in which a nose poke through the active hole had no consequences. Mice were considered to have acquired a stable self-administration behaviour when the following criteria were met for 2 consecutive FR1 sessions: (i) 80% stability in reinforcements (the number of reinforces on each day deviated by < 20% from the mean number of reinforces over the 2 consecutive days); (ii) ≥ 65% of responses were received at the active hole; and (iii) a minimum of 5 infusions per session. Mice that met the acquisition criteria were considered high-responders whereas mice that did not were considered low-responders for this task. All animals received a vehicle or CBD (20 mg/kg, i.p.) injection immediately before each session.

#### 2.4.3. Progressive Ratio Test

Mice underwent the progressive ratio (PR) test 24 h after the last day of acquisition. The PR test consisted of a single-session test that lasted 2 h. In this session, the requirement to earn an infusion escalated according to the following series: 1-2-3-5-12-18-27-40-60-90-135-200-300-450-675-1000. The breaking point is the last ratio reached by each animal, which is considered the highest effort the mouse makes to obtain an infusion. Mice received a vehicle or CBD (20 mg/kg, i.p.) injection immediately before the PR session.

### 2.5. Elevated Plus Maze (EPM)

The EPM was adapted from [38] to evaluate anxiety-like behaviour in mice. The apparatus (Panlab s.l.u., Barcelona, Spain) consisted of a black maze with four arms (16 × 5 cm) set in the form of a cross from a neutral central square (5 × 5 cm). Two arms were closed by vertical walls (closed arms) while the other two perpendicular arms had open edges (open arms). The maze stood at 30 cm above the floor in dim lighting conditions (30 lux). The percentage of time spent in the open arms was calculated by dividing the time spent in the open arms by the summation of the time spent in the open and closed arms. Mice received the CBD (20 mg/kg, i.p.) or vehicle injection 30 min before starting the test and the MDPV (3 or 4 mg/kg) or saline injection, 5 min before.

### 2.6. Statistical Analysis

Data are presented as mean ± SEM. For statistical analysis, we utilised GraphPad Prism 8.0. Software. For MDPV-induced CPP, we used a two-way ANOVA with session (test/pretest) and treatment (SAL-VEH/SAL-CBD/MDPV-VEH/MDPV-CBD) as factors. For nose poke activation in the self-administration experiments, we used a three-way ANOVA with repeated measures, with hole (active/inactive) and time (self-administration day) as within-subjects factors and CBD treatment (VEH/CBD) as between-subjects factor. To analyse the number of infusions in the self-administration experiments, we calculated a two-way ANOVA with repeated measures, with time (self-administration day) as a within-subjects factor and CBD treatment (VEH/CBD) as a between-subjects factor. We used two-way ANOVA for the analysis of the intake, with CBD treatment (VEH-CBD) and group (high-responders/low-responders) as factors, and progressive ratio, with CBD treatment (VEH/CBD) and hole (active/inactive) as factors. Unpaired Student’s *t*-tests were calculated for areas under the curve and breaking points. Finally, one-way ANOVA was calculated for the analysis of EPM with treatment (SAL-VEH/SAL-CBD/MDPV-VEH/MDPV-CBD) as a factor. When F achieved significance and there was no significant variance in homogeneity, Bonferroni’s post hoc test was run. The percentage of acquisition was analysed using Fisher’s exact test.

## 3. Results

### 3.1. CBD Treatment Partially Prevents MDPV-Induced CPP

**Figure 1:**
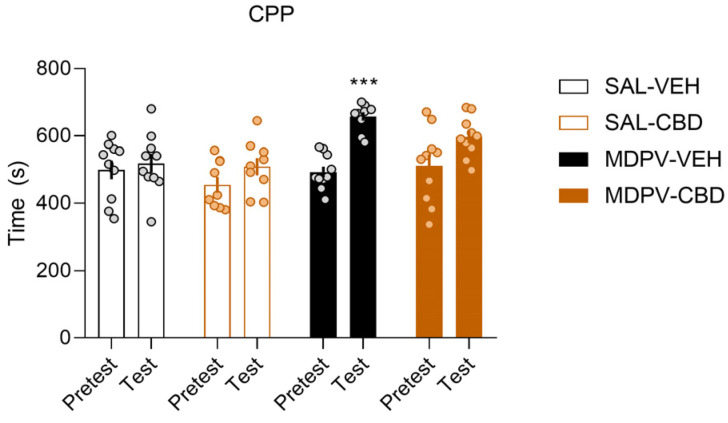
CBD slightly ameliorates MDPV (2 mg/kg)-induced CPP. (A) Time spent in the drug/saline-paired compartment during the pretest and test (*n*= 9–10/group). Two-way ANOVA, *** *p* < 0.001.

A two-way ANOVA for the time spent in the drug-paired compartment revealed an effect for session (Figure 1; F_(1,35)_ = 23.56, *p* < 0.001), treatment (F_(3,35)_ = 5.509, *p* < 0.01) and their interaction (F_(3,35)_ = 3.573, *p* < 0.05). Bonferroni’s post hoc analysis showed that only the MDPV-VEH group spent significantly more time in the drug-paired compartment during the test compared to the pretest (*p* < 0.001). However, the post hoc analysis also revealed that the MDPV-CBD group almost yielded significance (*p* = 0.052), indicating that CBD treatment mildly attenuated MDPV conditioning.

### 3.2. CBD Does Not Modify MDPV (0.05 mg/kg)-Induced Self-Administration

**Figure 2:**
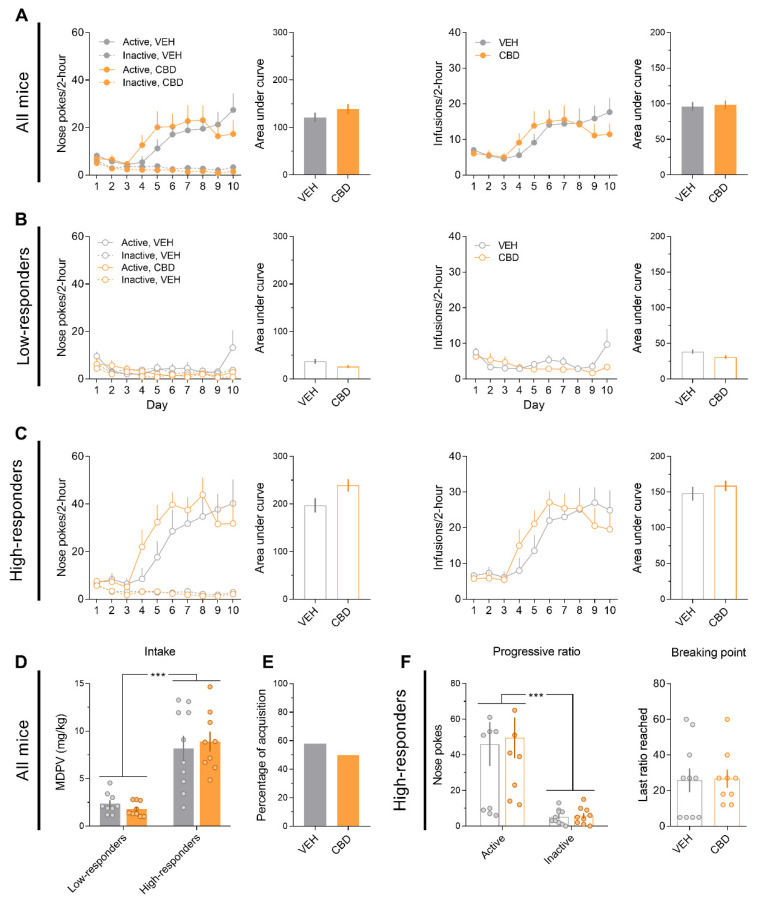
CBD does not modify MDPV (0.05 mg/kg/infusion) self-administration. (**A**) Nose pokes and infusions along the self-administration days and their respective areas under the curve (VEH, *n* = 19; CBD, *n* = 18). (**B**) Nose pokes and infusions along the self-administration days and their respective areas under the curve for low-responders (VEH, *n* = 9; CBD, *n* = 9). (**C**) Nose pokes and infusions along the self-administration days and their respective areas under the curve for high-responders (VEH, *n* = 10; CBD, *n* = 9). (**D**) Total MDPV intake. Two-way ANOVA, *** *p* < 0.001. (**E**) Percentage of acquisition. (**F**) Active/inactive nose pokes and breaking point during the progressive ratio test. Two-way ANOVA, *** *p* < 0.001.

Administration of CBD (20 mg/kg) during the acquisition of MDPV (0.05 mg/kg) self-administration did not modify drug-seeking or drug-taking behaviours (Figure 2A). Mice were later divided into low-responders (VEH, *n* = 9; CBD, *n* = 9) and high-responders (VEH, *n* = 10; CBD, *n* = 9) based on whether they met the acquisition criteria or not. Similarly, CBD did not affect any of these groups of mice (Figure 2B,C). A two-way ANOVA for total intake revealed that high responders consumed more MDPV than low-responders (Figure 2D; F_(1,33)_ = 50.11, *p* < 0.001), but no effects were found for CBD treatment or their interaction. The percentage of acquisition was not affected by the CBD treatment (Figure 2E).

Regarding the progressive ratio test, only high-responder mice underwent the progressive ratio test, since the MDPV-seeking behaviour of low-responders was not enough to meet the requirements of the task. The two-way ANOVA calculated for the nose pokes conducted during the progressive ratio test showed a significant effect for the factor hole (Figure 2F; F_(1,17)_ = 26.42, *p* < 0.001), but no effects were found for CBD treatment or their interaction. Moreover, the breaking point was not affected by CBD treatment (Figure 2F).

### 3.3. CBD Increases MDPV (0.075 mg/kg) Reinforcing Effects Only in High-Responders

**Figure 3:**
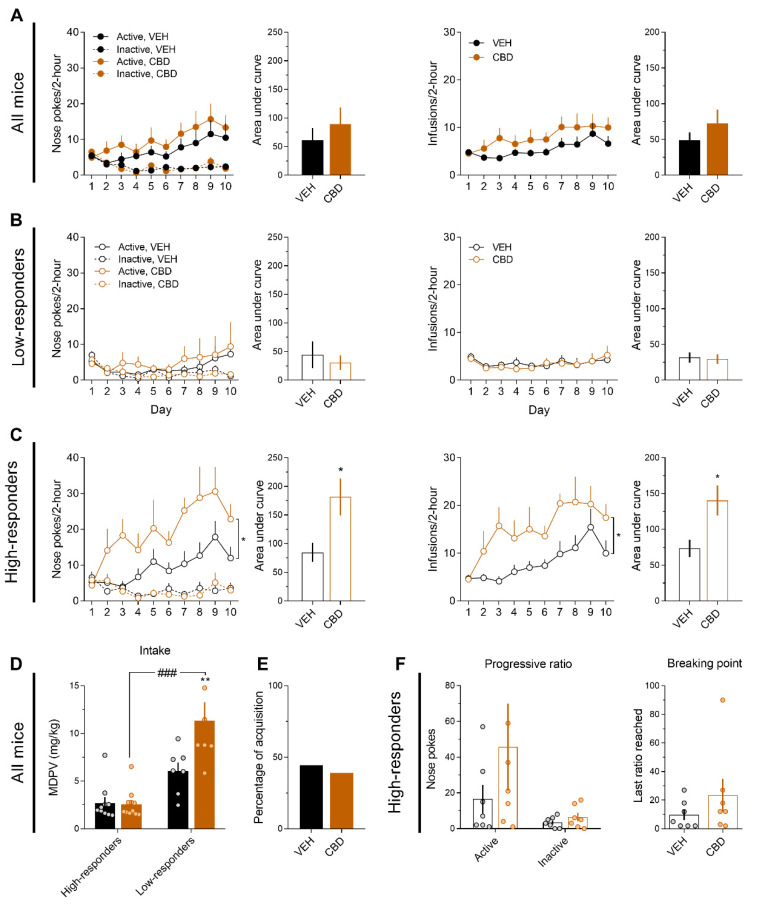
CBD increases MDPV (0.075 mg/kg/infusion) self-administration only for high responder mice. (**A**) Nose pokes and infusions along the self-administration days and their respective areas under the curve (VEH, *n* = 17; CBD, *n* = 18). (**B**) Nose pokes and infusions along the self-administration days and their respective areas under the curve for low-responders (VEH, *n* = 10; CBD, *n* = 11). (**C**) Nose pokes and infusions along the self-administration days and their respective areas under the curve for high-responders (VEH, *n* = 7; CBD, *n* = 7). Three-way ANOVA, * *p* < 0.05; Two-way ANOVA, * *p* < 0.05; Student’s t test, * *p* < 0.05. (**D**) Total MDPV intake. Bonferroni, ### *p* < 0.001, ** *p* < 0.01 vs vehicle low-responders. (**E**) Percentage of acquisition. (**F**) Active/inactive nose pokes and breaking point during the progressive ratio test.

Administration of CBD (20 mg/kg) during the acquisition of MDPV (0.075 mg/kg) self-administration does not modulate drug-seeking or drug-taking behaviours (Figure 3A). However, when data of low-responders (VEH, *n* = 10; CBD, *n* = 11) and high-responders (VEH, *n* = 7; CBD, *n* = 7) were analysed independently, we found statistical differences. CBD exerted no effect over low-responders’ behaviour (Figure 3B). However, for high responders (Figure 3C), the three-way ANOVA for the nose pokes curve revealed a significant effect of time (F_(9,108)_ = 4.29, *p* < 0.001), CBD treatment (F_(1,12)_ = 5.215, *p* < 0.05), hole (F_(1,12)_ = 29.64, *p* < 0.001), as well as the interactions time x hole (F_(9,108)_ = 6.336, *p* < 0.001) and CBD treatment x hole (F_(1,12)_ = 6.137, *p* < 0.05). Bonferroni’s post hoc for the CBD treatment x hole interaction revealed that CBD-treated group conducted more active nose pokes than the control group (*p* < 0.05), as expressed also by the area under the curve (Figure 3C; t_(12)_ = 2.67, *p* < 0.05). In the same line, the ANOVA for infusions revealed a significant effect of time (F_(9, 108)_ = 6.258, *p* < 0.001) and CBD treatment (F_(1,12)_ = 6.185, *p* < 0.05), with more infusions of the CBD-treated group. Again, the area under the curve was increased in CBD-treated mice (Figure 3C; t_(12)_ = 2.776, *p* < 0.05).

A two-way ANOVA for total intake (Figure 3D) revealed significant effects of CBD treatment (F_(1,31)_ = 39.04, *p* < 0.001), group (high vs low-responders; F_(1,31)_ = 7.092, *p* < 0.05) and their interaction (F_(1,31)_ = 7.874, *p* < 0.01). Bonferroni’s post hoc showed that CBD-treated high-responder mice consumed more than vehicle-treated high-responders (*p* < 0.01) and more than CBD-treated low-responders (*p* < 0.001). The percentage of acquisition was not affected by the CBD treatment (Figure 3E).

Again, only high-responder mice underwent the progressive ratio test. A two-way ANOVA for the nose pokes conducted during the progressive ratio test revealed a tendency for the factor hole (F_(1,12)_ = 4.617, *p* = 0.052), but no effects of CBD treatment or their interaction. Moreover, the breaking point was not affected by CBD treatment.

### 3.4. Mice Modulate Their Drug-Seeking Behaviour to Maintain MDPV Consumption

**Figure 4:**
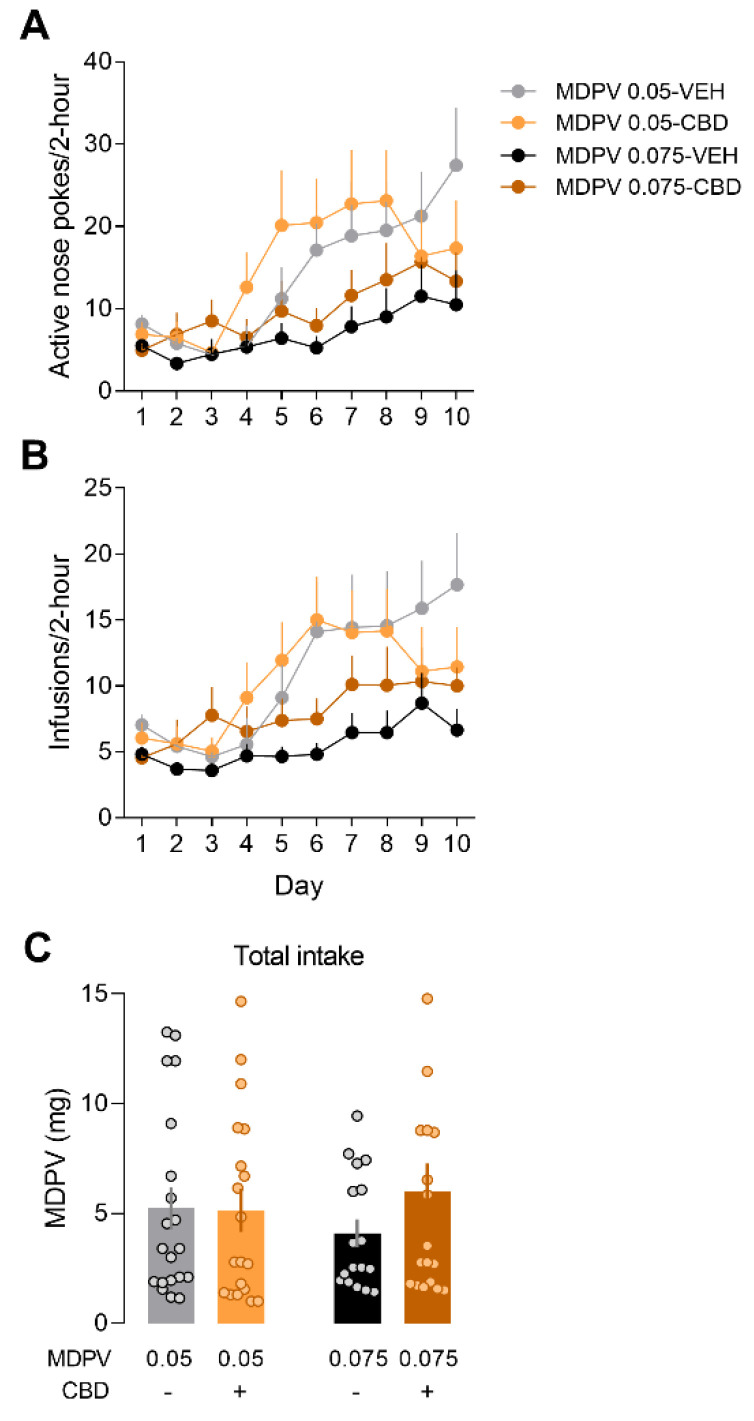
Mice modulate their drug-seeking behaviour to maintain MDPV consumption. (**A**) Active nose pokes and (**B**) infusions along the self-administration. (**C**) Total intake of MDPV.

CBD effects on drug-seeking and taking behaviours for both doses of MDPV (0.05 and 0.075 mg/kg/infusion) were analysed for all mice (high- and low-responders). A repeated measures three-way ANOVA for the active nose pokes (Figure 4A) revealed a significant effect of time (F_(9,612)_ = 9.969, *p* < 0.001), MDPV dose (F_(1,68)_ = 4.841, *p* < 0.05) and their interaction (F_(9,612)_ = 2.595, *p* < 0.01). No effects of CBD or any interaction with it were found. Bonferroni’s post hoc for the interaction MDPV dose x time revealed that mice self-administering MDPV 0.05 mg/kg/infusion conducted more active responses on days 1 (*p* < 0.05), 6 (*p* < 0.01) and 7 (*p* < 0.05) compared to those self-administering MDPV 0.075 mg/kg/infusion. For the infusions curve (Figure 4B), the factors time (F_(9,612)_ = 9.837, *p* < 0.001), MDPV dose (F_(1,68)_ = 5.285, *p* < 0.05) and their interaction (F_(9,612)_ = 2.623, *p* < 0.01) reached significance. Bonferroni’s post hoc for the interaction MDPV dose x time revealed that mice self-administering MDPV 0.05 mg/kg/infusion received more infusions on days 1 (*p* < 0.05), 6 (*p* < 0.01), 7 (*p* < 0.05) and 10 (*p* < 0.05) compared to those self-administering MDPV 0.075 mg/kg/infusion. Again, no effects of CBD or any interaction with it were found. The analysis of the total intake (Figure 4C) revealed no significant differences due to the MDPV dose or CBD treatment, indicating that mice consumed the same amount of MDPV regardless of the experimental conditions.

### 3.5. CBD Increases the Anxiolytic Effects of MDPV in the EPM

**Figure 5:**
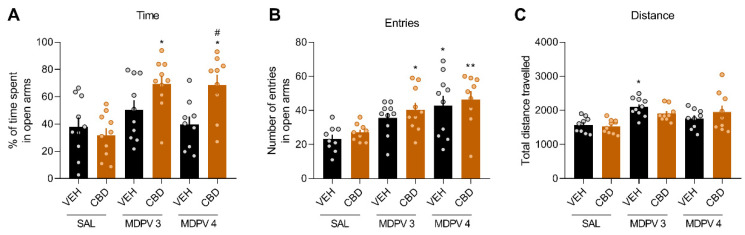
CBD exerts anxiolytic effects in the EPM only when co-administered with MDPV. (**A**) Percentage of time spent in open arms (*n*= 9–10/group). Bonferroni, * *p* < 0.05 vs VEH-SAL, # *p* < 0.05 vs VEH-MDPV 4 mg/kg. (**B**) Number of entries in open arms. Bonferroni, * *p* < 0.05 vs VEH-SAL, ** *p* < 0.01 vs VEH-SAL. (**C**) Total distance travelled. Bonferroni, * *p* < 0.05 vs VEH-SAL.

One-way ANOVA for the percentage of time spent in open arms (Figure 5A) revealed a significant effect of treatment (F_(2,52)_ = 6.304, *p* < 0.001). Bonferroni’s post hoc analysis indicated that CBD treatment increased the percentage of time spent in open arms when administered to the MDPV 3 and 4 mg/kg mice compared to the VEH-SAL (*p* < 0.05) and VEH-CBD (*p* < 0.01) groups. Moreover, mice that received both MDPV 4 mg/kg and CBD displayed increased percentage of time spent in open arms compared to those that only received MDPV 4 mg/kg (*p* < 0.05). In addition, the one-way ANOVA for the number of entries in open arms revealed a significant effect of treatment (Figure 5B; F_(2,51)_ = 5.04, *p* < 0.001). The post hoc analysis indicated that the number of entries in open arms was increased in the CBD-MDPV 3 mg/kg (*p* < 0.05), VEH-MDPV 4 mg/kg (*p* < 0.05) and CBD-MDPV 4 mg/kg (*p* < 0.01) groups compared to the VEH-SAL group. Moreover, the number of entries in open arms was also increased in the CBD-MDPV 4 mg/kg group compared to the CBD-SAL group. Finally, the one-way ANOVA for total distance revealed a significant effect of treatment (Figure 5C; F_(5,50)_ = 4.65, *p* < 0.01). The post hoc analysis of MDPV dose indicated that mice treated with MDPV 3 mg/kg and vehicle traversed more distance compared to the VEH-SAL group (*p* < 0.05).

## 4. Discussion

In the present report, we evaluated the impact of CBD (20 mg/kg) on MDPV motivational effects in mice. The current results provide evidence of the mild mitigation of MDPV-induced CPP due to CBD treatment. On the contrary, CBD administration throughout MDPV self-administration increased drug-seeking and taking behaviours, but only in the high-responders group of mice with an MDPV dose of 0.075 mg/kg/infusion. Additionally, we observed that acute administration of CBD increased the anxiolytic-like effects elicited by MDPV.

Previous research has evidenced that MDPV induces CPP at doses similar to those used in this study in both rats [42] and mice [11]. Here, we confirmed the expression of CPP induced by MDPV 2 mg/kg, i.p. However, the CBD effects on this paradigm only showed a mild attenuation of MDPV’s rewarding effects. We previously reported that CBD pretreatment (10 and 20 mg/kg) decreased the acquisition of cocaine-induced CPP [33]. Nevertheless, recent studies using more similar approaches (that is, CBD treatment during cocaine-induced CPP conditioning) did not reveal a significant effect of CBD administration at either 10 mg/kg [43] or CBD 30 or 60 mg/kg [44]. Similarly, CBD (5 mg/kg) administered during the conditioning phase did not modulate the expression of amphetamine-induced place preference [36]. Taken together, these data suggest that, except in rare cases, CBD does not affect the rewarding effects of psychostimulants. Moreover, CBD modulation of the CPP paradigm is highly dependent on the psychostimulant used, as supported by previous studies highlighting the behavioural and molecular differences between cocaine and MDPV [11,45,46].

As mentioned above, MDPV serves as a reinforcer during the intravenous self-administration paradigm in rats [47]. In the present study, MDPV at doses of 0.05 and 0.075 mg/kg/infusion induced similar levels of drug intake in the self-administration paradigm. Therefore, mice receiving the lower dose of MDPV increased the number of nose pokes and infusions to obtain the same amount of drug as mice receiving the higher dose of the psychostimulant. Under our experimental conditions, around 50% of mice showed acquisition, in contrast with previous studies in which the acquisition percentages in rats rounded up to 80% [19]. These differences might be due to methodological variations (i.e., acquisition criteria, duration of the sessions, days of self-administration, MDPV doses, etc.) or to species-dependent changes in the response to MDPV. In this sense, previous work in our laboratory using other psychostimulants as reinforcers reached variable rates of acquisition depending on the drug, that is, 80–90% for cocaine [33] and 57–67% for 3,4-methylenedioxymethamphetamine (MDMA) [48]. However, studies using rats [9,21,49] and rhesus monkeys [50] suggest that MDPV functions as a more effective reinforcer than cocaine. Therefore, this evidence hints at a more effective role of MDPV in inducing reinforcing effects in the self-administration paradigm in rats compared to mice.

CBD administered during the acquisition of self-administration did not modify MDPV intake at any of the tested doses. However, due to the high variability observed in mice’s response to MDPV (around 50% reaching acquisition criteria regardless of the dose), we decided to split the group of animals into high- and low-responders for the analysis, to better elucidate CBD effects over these two groups. Previous studies have already evidenced the existence of low- and high-responder rats regarding the relative reinforcing effects of MDPV [21,51], albeit they used a different criterion for dividing these populations. Surprisingly, although CBD did not modify the percentage of mice reaching the acquisition criteria regardless of the drug dose, CBD modulation of MDPV self-administration was very different in the two populations for the dose 0.075 mg/kg/infusion. While low-responders were unaffected by CBD, high-responders increased drug-taking behaviour after CBD treatment. Therefore, low- and high-responders not only differ in their response to MDPV, but also in the effects that CBD exerts over it when a higher dose of MDPV is used as a reinforcer.

Previous research has reported that CBD (20 mg/kg) treatment during self-administration decreases the reinforcing effects of natural rewards, such as sucrose [52], and cocaine [33,34,53]. However, the motivation to self-administer methamphetamine in a progressive ratio test was only reduced with a high dose of CBD (80 mg/kg) [37]. This evidence is contrary to the results obtained in this study, where CBD had no effects over MDPV self-administration (and progressive ratio) or even increased it. Several explanations could be responsible for these differential outcomes. First, CBD, at other doses or different treatment schedules, could have resulted in different outcomes among the groups. Second, MDPV differs from the psychostimulants mentioned above in its potency and targets within the brain [4,7,54] as well as in its behavioural and cognitive effects [8,45]. Therefore, it is reasonable to consider that the interactions between CBD and MDPV are different from the interactions with other psychostimulants. Third, MDPV can exert aversive effects, notably at high doses, as measured in the conditioned taste aversion test [42,55,56]. In addition, the anxiolytic effects of CBD have been also demonstrated [33,57]. These observations support the hypothesis that CBD could be mitigating the aversive effects of MDPV providing a better “environment” for the operant task in the self-administration paradigm. This hypothesis would be following the fact that CBD only increased MDPV self-administration in the high-responders and at the highest dose of MDPV.

Finally, we assessed the effects of acute MDPV treatment at doses that are shown to cause aversion in rats [42,55,56] on anxiety-like behaviour and we co-administered CBD to test the possible interactions. Interestingly, we observed that acute administration of CBD exerted anxiolytic-like effects, but only when co-administered with MDPV. Although MDPV increased the number of entries in open arms, this is probably due to the increase in locomotor activity rather than an anxiolytic effect itself. Otherwise, CBD did not modify the number of entries in open arms or the total distance travelled, indicating that its anxiolytic effect was not due to alterations in the locomotor activity. Moreover, CBD had no effects when administered acutely to the vehicle group, which means that the anxiolytic-like effect was due to an interaction with MDPV. This lack of effect of CBD acute administration in the EPM is in line with a previous study using the same dose [58] in rats. However, a different study with mice as experimental subjects found an anxiolytic effect of acute administration of CBD with doses ranging from 0.5 to 50 mg/kg [59]. To the best of our knowledge, this is the first study examining the effects of MDPV acute administration on anxiety-like behaviour, as well as the CBD modulation. Taking this evidence into account, it is reasonable to propose that such anxiolytic effects arising from the CBD and MDPV synergy could be contributing to the CBD-induced increase in MDPV reinforcement. Therefore, more research is needed to fully understand the interaction between these compounds and to shed some light on the molecular mechanisms responsible for such effects.

On this basis, our results suggest that CBD modulation of MDPV-induced responses in mice is very complex and varies depending on the requirements of the task. First, the results obtained from the CPP suggest mild CBD-induced mitigation of MDPV’s rewarding effects. However, the results from the self-administration paradigm indicate that CBD might be potentiating the reinforcing effects of MDPV. In the same line, CBD also seems to exert anxiolytic effects in combination with MDPV in the EPM paradigm. Given the considerable preclinical evidence supporting the protective role of CBD in psychostimulant addictive-like behaviours together with the results obtained in this study, we conclude that further research attempting to decipher the behavioural and molecular interactions between CBD and MDPV is needed.

## Data Availability

Not applicable.

## Abbreviations

CBD	cannabidiol
CPP	conditioned place preference
DAT	dopamine transporter
EPM	elevated plus maze
FR1	fixed ratio 1
MDMA	3:4-methylenedioxymethamphetamine
MDPV	3:4-methylenedioxypyrovalerone
NET	norepinephrine transporter
NPS	new psychoactive substances
PR	progressive ratio
SERT	serotonin transporter
